# Tofacitinib-Associated Acne in a Young Patient With Severe Atopic Dermatitis

**DOI:** 10.7759/cureus.115307

**Published:** 2026-08-27

**Authors:** Sabrina Oujdi, Ouiame El Jouari, Salim Gallouj

**Affiliations:** 1 Dermatology, Centre Hospitalier Universitaire Mohammed VI, Tangier, MAR

**Keywords:** acne, adverse drug reaction, atopic dermatitis, inflammatory acne, janus kinase inhibitors, tofacitinib

## Abstract

Atopic dermatitis (AD) is a chronic inflammatory skin disease that may require systemic therapy in patients with moderate-to-severe or refractory disease. Tofacitinib, a Janus kinase (JAK) inhibitor, has demonstrated efficacy in the treatment of severe inflammatory dermatoses, including refractory AD. However, its use may be associated with various adverse events, and acneiform eruptions have increasingly been reported with JAK inhibitor therapy.

We report the case of an 18-year-old female patient with severe AD who was treated with tofacitinib, resulting in marked improvement of her eczematous lesions and pruritus. After several weeks of treatment, she developed inflammatory facial acne without a history of severe acne or any other identified precipitating factor. Clinical examination revealed multiple inflammatory papules and pustules predominantly involving the centrofacial region, associated with comedones and microcysts. Laboratory investigations were unremarkable. The acne was successfully managed with appropriate topical therapy while tofacitinib was continued.

This case highlights acne as a potentially emerging cutaneous adverse event associated with JAK inhibitor therapy. Early recognition and appropriate management may help prevent impairment of quality of life and treatment adherence while allowing continuation of effective systemic therapy for severe AD.

## Introduction

Atopic dermatitis (AD) is a common chronic inflammatory skin disease resulting from complex interactions between epidermal barrier dysfunction, genetic susceptibility, environmental factors, and immune dysregulation, particularly involving type 2 cytokine pathways. Moderate-to-severe AD can substantially impair quality of life and may require systemic therapy when conventional topical treatments and phototherapy are insufficient. The recognition of the central role of the Janus kinase/signal transducer and activator of transcription (JAK/STAT) pathway in AD pathogenesis has led to the development of targeted therapies, including JAK inhibitors [1,2].

Tofacitinib is an oral JAK inhibitor with preferential activity against JAK1 and JAK3. It has demonstrated efficacy in several chronic inflammatory diseases and has also shown promising results in severe and refractory AD. Its therapeutic effect is mediated by inhibition of signaling downstream of several cytokines involved in cutaneous inflammation, resulting in improvement of inflammatory skin lesions and pruritus [3,4].

With the increasing use of JAK inhibitors in dermatology, new cutaneous adverse events have been recognized. Among these, acne has emerged as a potentially underrecognized adverse event. A recent meta-analysis of phase II and III clinical trials of JAK inhibitors demonstrated a significantly increased risk of acne among treated patients, particularly in dermatological indications. Several studies involving patients with AD have further supported this association, with acne reported among the most frequent cutaneous adverse events with certain agents in this therapeutic class. Although acne associated with JAK inhibitors is generally mild to moderate in severity, its underlying pathophysiological mechanisms remain poorly understood. Its clinical impact may be particularly relevant in adolescents and young adults. Data specifically addressing tofacitinib-associated acne remain limited, as most published studies have focused on second-generation JAK inhibitors.

We report the case of an 18-year-old female patient with severe atopic dermatitis who developed inflammatory facial acne characterized by both inflammatory and retention lesions, including open and closed comedones, distinguishing it from the acneiform eruptions commonly associated with this class of treatment. This case adds to the growing body of evidence regarding this potentially emerging cutaneous adverse event by highlighting the occurrence of mixed acne following systemic treatment with a JAK1/JAK3 inhibitor

## Case presentation

An 18-year-old female patient had been followed for severe AD since the age of four years. Her disease had been managed with topical therapies and phototherapy, with limited clinical improvement and substantial impairment of quality of life. Because of persistent severe disease despite conventional treatment, systemic therapy with tofacitinib at a dose of five mg once daily was initiated.

After three months of treatment, the patient experienced marked improvement of her AD, with substantial regression of eczematous plaques and significant reduction in pruritus. However, during the course of treatment, she developed new inflammatory lesions on the face. The patient had no history of severe acne before initiation of tofacitinib, and no other apparent precipitating factor was identified.

**Figure 1:**
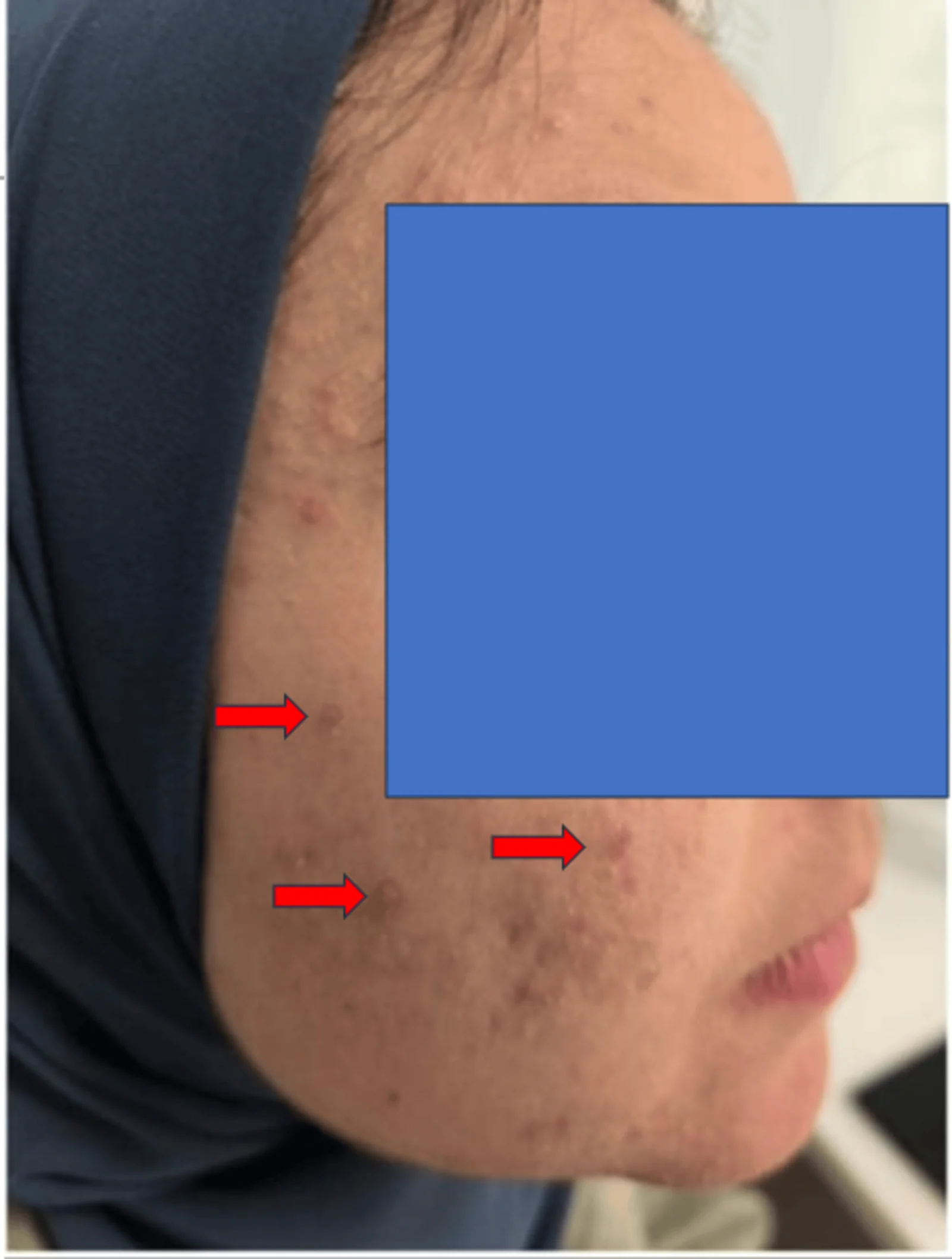
Clinical image showing mixed facial acne characterized by multiple erythematous inflammatory papules and pustules associated with open and closed comedones, predominantly distributed over the right cheek

**Figure 2:**
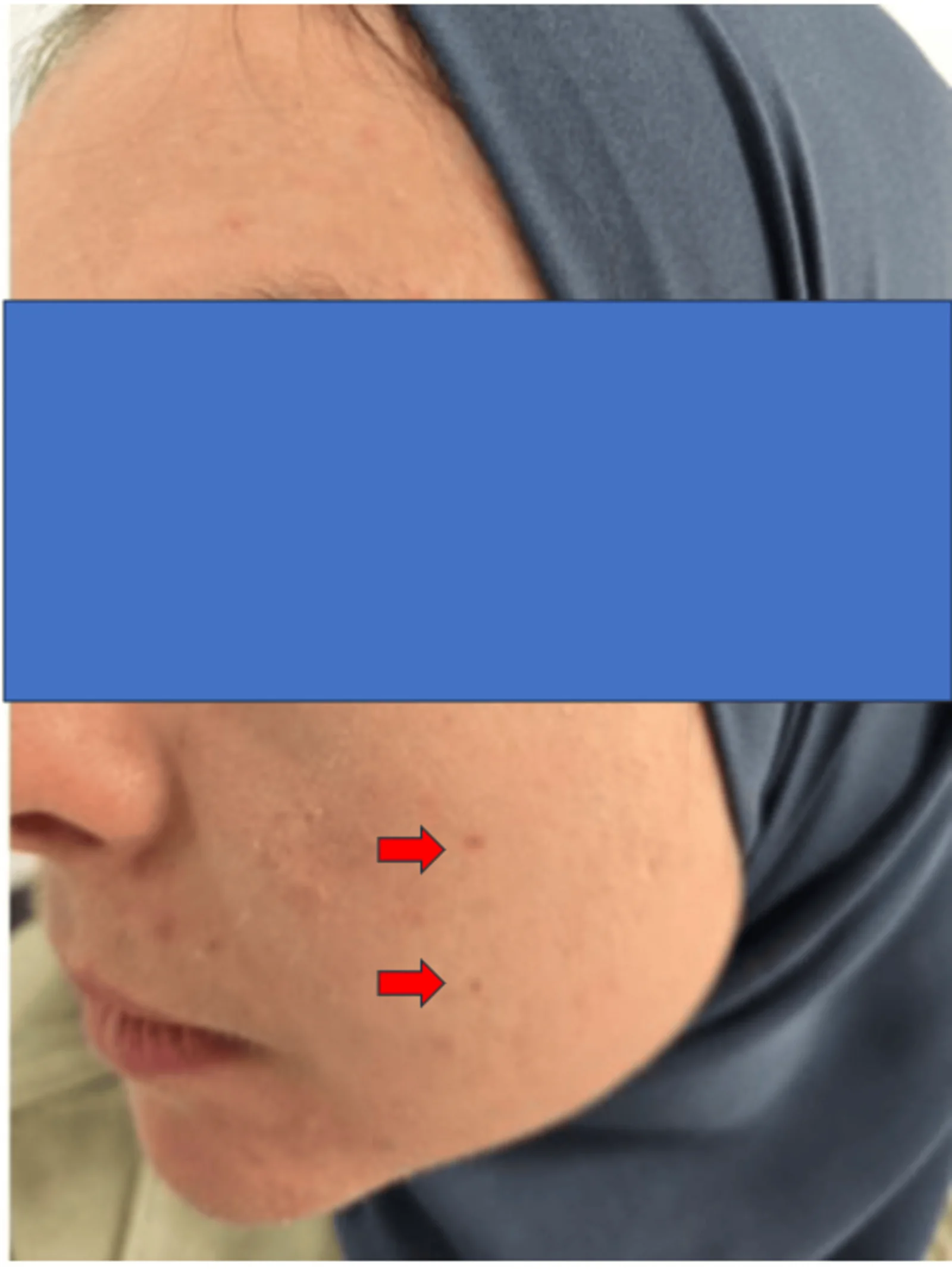
Clinical image showing mixed facial acne characterized by multiple erythematous inflammatory papules and pustules associated with open and closed comedones, predominantly distributed over the left cheek

Dermatological examination revealed multiple erythematous inflammatory papules and pustules involving both cheeks and the forehead, associated with microcomedones and open comedones. The clinical presentation was consistent with mixed facial acne (Figures 1, 2).

No other significant cutaneous or systemic abnormalities were identified. In particular, the patient had no fever or respiratory, gastrointestinal, neurological, abdominal, or urinary symptoms. The diagnosis of mixed acne developing during tofacitinib therapy was established clinically. Laboratory investigations performed during follow-up were unremarkable.

Given the temporal association between the initiation of tofacitinib and the development of acne, the absence of a previous history of severe acne, and the absence of another identifiable precipitating factor, a possible association between tofacitinib therapy and the development of acne was considered.

The patient was treated with topical adapalene 0.1% and benzoyl peroxide 2.5% in the evening, together with appropriate photoprotection. This treatment was administered for 3 months, resulting in marked improvement of both inflammatory and comedonal lesions. Maintenance therapy with the same treatment was then continued twice weekly throughout the duration of tofacitinib therapy (Table 1).

**Table 1:** Clinical course, treatment, and outcome of tofacitinib-associated mixed facial acne

Time point	Atopic dermatitis status	Acne clinical presentation	Treatment	Clinical outcome
Baseline	Severe atopic dermatitis	No acne lesions reported	Tofacitinib initiated	Improvement in atopic dermatitis
3 months after starting tofacitinib	Controlled/improved atopic dermatitis	Mixed facial acne with inflammatory papules and pustules associated with open and closed comedones	Topical anti-acne treatment initiated	—
After 3 months of topical treatment	Stable atopic dermatitis	Marked regression of inflammatory and comedonal lesions	Same topical treatment continued twice weekly as maintenance therapy	Favorable clinical response
Follow-up during tofacitinib therapy	Maintained clinical control	No significant recurrence of acne reported	Maintenance topical therapy twice weekly	Sustained improvement

## Discussion

Tofacitinib is an oral Janus kinase (JAK) inhibitor that preferentially inhibits JAK1 and JAK3 and also exhibits activity against JAK2. By interfering with the JAK-STAT signaling pathway, it modulates several cytokine pathways involved in inflammatory dermatoses. Although its use in atopic dermatitis (AD) has been investigated in clinical trials, tofacitinib is not currently among the main approved systemic treatments for AD in many countries. Nevertheless, clinical improvement has been reported in patients with severe or refractory disease treated with this drug [2,4].

The increasing use of JAK inhibitors in dermatology has highlighted several cutaneous adverse events, including acne and acneiform eruptions. Acne represents a potentially important adverse event associated with this therapeutic class, particularly in patients treated for dermatological conditions. In a systematic review and meta-analysis of 25 phase II and III randomized controlled trials involving 10,839 participants, Martinez et al. found that treatment with JAK inhibitors was associated with a significantly increased risk of acne compared with placebo, with an overall odds ratio of 3.83 (95% CI, 2.76-5.32) [5]. This association was particularly pronounced in dermatological indications and with selective JAK1 inhibitors. These findings highlight the need to inform patients about the potential risk of acne before initiating JAK inhibitor therapy.

Data specifically concerning patients with atopic dermatitis (AD) are consistent with these findings. A systematic review and meta-analysis conducted by Sun et al., including 10 randomized controlled trials and 7,901 patients with AD, demonstrated an increased risk of incident acne among patients treated with systemic JAK inhibitors [6]. A subsequent systematic review and network meta-analysis of 24 randomized controlled trials in dermatology, including 11,396 patients, also confirmed an increased risk of acne associated with JAK inhibitors, with this association being particularly pronounced in patients with AD treated with selective JAK1 inhibitors [7]. These findings suggest that acne may represent a clinically relevant adverse event of JAK inhibition in dermatology, although the magnitude of the risk may vary according to the specific agent, dose, and treated population.

The clinical characteristics of JAK inhibitor-associated acne have been particularly well described with upadacitinib. In an integrated post hoc analysis of three phase III randomized controlled trials, Mendes-Bastos et al. reported acne in 9.8% of patients receiving upadacitinib 15 mg and in 15.2% of those receiving 30 mg, compared with 2.2% of patients receiving placebo during the controlled treatment period [8]. Acne occurred predominantly in younger patients and was generally mild to moderate in severity. Lesions mainly affected the face and were usually managed with conventional anti-acne treatments without requiring discontinuation of systemic therapy.

Similarly, Lee et al. described eight patients with atopic dermatitis who developed acne during treatment with systemic JAK inhibitors, primarily upadacitinib and baricitinib [9]. In this series, acne generally developed within the first few months of treatment and predominantly involved the face. The authors noted that most cases could be managed with conventional topical acne therapy while continuing the JAK inhibitor. These observations are relevant to our patient, in whom inflammatory facial acne developed after initiation of tofacitinib, while her underlying atopic dermatitis showed marked improvement.

In contrast to the growing body of evidence regarding upadacitinib and other selective JAK1 inhibitors, published data specifically describing tofacitinib-associated acne remain limited. A case report by Biswal et al. described the development of acne in a patient with vitiligo vulgaris treated with tofacitinib, providing additional evidence suggesting that acne may occur during treatment with this agent [10]. However, isolated cases cannot establish causality, and the available data remain insufficient to determine the incidence or relative risk of acne specifically associated with tofacitinib.

In our patient, several clinical features suggest a possible association between tofacitinib and the development of acne. First, the patient had no history of severe acne prior to treatment. Second, the lesions developed after initiation of tofacitinib, while her atopic dermatitis was showing marked improvement. Third, no other obvious triggering factor was identified. Finally, the acne improved after initiation of topical anti-acne therapy while tofacitinib was continued. Although these observations do not establish a causal relationship, the temporal sequence and clinical course are compatible with a possible drug-related adverse event.

The underlying pathophysiological mechanisms of JAK inhibitor-associated acne remain poorly understood. The JAK-STAT pathway is involved in numerous processes regulating inflammatory signaling, keratinocyte biology, and immune responses. Alterations in these pathways following pharmacological JAK inhibition could potentially modify the inflammatory environment of the pilosebaceous unit. However, the exact mechanisms leading to acne during JAK inhibitor therapy remain uncertain and are likely multifactorial [9].

One possible mechanism involves alterations in the skin microbiome. Recent work by Wang et al. demonstrated proliferation of Cutibacterium acnes in patients treated with JAK inhibitors, providing a potential mechanistic link between JAK inhibition and the development of acneiform eruptions [11]. This observation is particularly relevant because C. acnes plays an important role in acne pathogenesis through interactions with the follicular microenvironment and activation of innate inflammatory pathways. However, whether this microbial alteration directly contributes to acne during JAK inhibition remains to be determined.

The relationship between JAK signaling and acne is complex. JAK-STAT pathway activation has itself been demonstrated in acne lesions, suggesting that JAK signaling participates in inflammatory processes within acne [12]. Therefore, the development of acne during JAK inhibitor therapy cannot simply be explained by generalized immune activation. Instead, it may result from a complex interaction between cytokine signaling, follicular keratinization, sebaceous gland activity, immune modulation, and changes in the skin microbiome.

Patient characteristics may also influence the risk of acne. Clinical studies of upadacitinib have suggested that younger age and female sex may be associated with a higher frequency of acne [8,13]. In the phase III Rising Up study, acne was reported more frequently with the higher dose of upadacitinib, and the majority of cases were mild or moderate and managed with topical therapy [13]. These findings are particularly relevant to our patient, who was an 18-year-old female.

From a therapeutic perspective, the occurrence of acne does not necessarily require discontinuation of an effective JAK inhibitor. Most reported cases have been mild to moderate and have responded to conventional topical acne therapies, including topical retinoids and benzoyl peroxide [8,9,13]. In our patient, treatment with adapalene 0.1% and benzoyl peroxide 2.5% was initiated while tofacitinib was maintained because of its substantial efficacy in controlling severe AD. This approach allowed simultaneous management of the adverse event and preservation of the clinical benefit of systemic therapy.

The decision to continue a JAK inhibitor should nevertheless be individualized. Severe acne, extensive disease, scarring, or significant psychological impairment may require more intensive treatment and, in selected cases, reconsideration of the systemic therapy. This is particularly important in adolescents and young adults, for whom facial acne may have a considerable impact on self-esteem and quality of life [14,15].

Our case has several limitations. First, the observation of a single patient cannot establish a definitive causal relationship between tofacitinib and acne. Second, no rechallenge was performed because re-exposure solely for diagnostic purposes would not have been justified. Third, no specific microbiological or mechanistic investigations were performed. Nevertheless, the absence of a history of severe acne, the temporal association with tofacitinib initiation, the characteristic clinical presentation, and the favorable response to topical therapy while continuing tofacitinib support the possibility of a treatment-associated event.

This case is noteworthy because reports specifically describing acne during tofacitinib therapy in patients with severe atopic dermatitis remain scarce compared with the growing literature concerning selective JAK1 inhibitors. Recent evidence suggests that JAK inhibitor-associated acneiform eruptions may involve alterations in the sebaceous microenvironment and expansion of Cutibacterium acnes, providing a potential mechanistic explanation for this adverse event [16]. The role of JAK-STAT signaling in atopic dermatitis and its modulation by JAK inhibitors may also contribute to changes in cutaneous immune homeostasis [17].

It also highlights an important practical point: the development of acne should not automatically lead to discontinuation of an otherwise effective JAK inhibitor. Early recognition and appropriate dermatological management may allow continuation of systemic treatment while preserving control of the underlying inflammatory disease. The pathogenesis of acne is multifactorial and involves sebaceous gland activity, follicular hyperkeratinization, C. acnes, and inflammation; therefore, targeted dermatological treatment may be sufficient in mild-to-moderate cases [15].

Further pharmacovigilance studies and prospective cohorts are required to determine the incidence of acne specifically associated with tofacitinib, identify potential risk factors, and clarify the biological mechanisms underlying JAK inhibitor-associated acne. Recent observations of C. acnes expansion during JAK inhibitor therapy provide a promising avenue for future mechanistic research [16]. Such data would help clinicians better counsel patients and develop standardized strategies for managing this potentially important adverse event.

## Conclusions

Tofacitinib-associated acne may represent a potential cutaneous adverse event that remains insufficiently characterized in patients with severe atopic dermatitis. Our case illustrates the development of mixed facial acne in a young patient three months after initiation of tofacitinib, suggesting a possible temporal association; however, a causal relationship cannot be established based on a single case. Although this adverse event appears to be generally mild to moderate in severity and may not require treatment discontinuation, it can negatively affect quality of life and treatment adherence, particularly among adolescents and young adults. Early recognition and appropriate management may allow continued tofacitinib therapy while preserving its therapeutic benefit for atopic dermatitis. Further studies are needed to better characterize the frequency and clinical spectrum of acne associated with JAK inhibitors, elucidate its underlying pathophysiological mechanisms, identify potential risk factors, and establish optimal management strategies
